# Mental Health and Substance Use in Rural and Northern British Columbia: A Systematic Review of Prevalence, Risk Factors, and Intervention Effectiveness

**DOI:** 10.7759/cureus.112169

**Published:** 2026-07-06

**Authors:** Cosmas C Ofoegbu, Chioma G Muoghalu, Samuel C Uwanna, Abimbola O Oladeji

**Affiliations:** 1 Community and Family Medicine, Fort Saint John Hospital, Fort St. John, CAN; 2 Health Sciences, Central Washington College, Enugu, NGA; 3 Medicine, University of Galway, Galway, IRL; 4 Medical Education, NYC Health + Hospitals/Harlem, New York, USA; 5 Medical Education, University of British Columbia, Vancouver, CAN

**Keywords:** british columbia, health disparities, mental health outcomes, rural and northern population, substance use disorder

## Abstract

The rural and northern communities of British Columbia (BC) have their own barriers to accessing mental health and substance use services that lead to greater susceptibility to psychological disorders and substance abuse. Although there is increasing awareness about these inequalities, the research on mental health and substance use outcomes in these groups is still piecemeal. The proposed systematic review provided a synthesis of the current literature on mental health and substance use outcomes in rural and northern BC, the population-specific risk and protective factors, and the effectiveness of planned interventions to address the identified issues. Quantitative studies published in peer-reviewed journals, mixed-methods studies, and government and other health reports published in English were also considered. Predefined terms were used to search databases such as PubMed, PsycINFO, Cumulative Index to Nursing and Allied Health Literature (CINAHL), Scopus, and Web of Science, as well as grey literature sources, for mental health, substance use, rurality, and BC. Two independent reviewers performed title/abstract and full-text screening, and any discrepancies were settled by consensus. A standardized form was used to extract data, focusing on the characteristics of the studies, prevalence, and types of mental health and substance use outcomes, risk and protective factors, and intervention outcomes. Quality checking was conducted using the Newcastle-Ottawa Scale for observational studies, the Critical Appraisal Skills Programme (CASP) Qualitative Studies Checklist for the qualitative studies, and the National Institutes of Health (NIH) Quality Assessment Tool for before-after (pre-post) studies with no control group. Primary evidence suggests that depression, anxiety, and substance use disorders are higher in rural and northern BC due to the lack of socioeconomic and service access and geographic isolation. The available evidence of interventions is disparate and insufficient, and suggests gaps in specific mental health and substance use interventions. The combination of these results can be a basis for policy formulation and distribution of resources, as well as culturally sensitive interventions to decrease mental health and substance use disparities within underserved BC groups.

## Introduction and background

Mental health and substance use disorders are an important public health problem in Canada and a significant cause of morbidity, disability, diminished quality of life, and early death. These conditions impact all populations in all geographic settings, but the burden may be higher in rural and remote areas where access to healthcare services is restricted, and social, economic, and environmental disadvantages are more pronounced [1]. The impact of geographic isolation, income and poverty, workforce shortages, transportation restrictions, and inequities in access to specialized care are complex factors contributing to rural health disparities. The understanding of how these factors shape mental health and substance use outcomes is critical to effective and equitable health policies.

These challenges can be explored in an important setting in Northern British Columbia (BC). Covering a vast geographic area characterized by dispersed populations, remote communities, and long travel distances between population centers, Northern BC faces unique healthcare delivery challenges [2]. The region is largely covered by Northern Health, one of BC's five regional health authorities, which provides healthcare services to a relatively small percentage of the population in a large area of the province [3]. There is a lack of mental health providers, addiction specialists, and primary healthcare providers in many communities, and this often leads to delayed diagnosis, treatment, and continued care. Northern BC also has a significant Indigenous population, including many First Nations communities, many of which continue to suffer from the legacy of colonization, intergenerational trauma, systemic discrimination, and unequal access to healthcare services [4].

The burden of mental health and substance use disorders in Northern BC is substantial. Higher than provincial rates of depression, anxiety, and psychological distress, substance-related harms, and suicide-related outcomes have been reported at the provincial/regional level, as compared to provincial/regional averages [5]. Access to mental health and addiction services is often difficult for those living in rural and remote communities, including transportation challenges, financial issues, a limited workforce, and limited treatment programs. These barriers may worsen health outcomes and health inequities for vulnerable populations, as well as delay care [6].

One of the major public health concerns in BC is the current toxic drug crisis. In April 2016, the Province of BC declared a public health emergency due to the surge in overdose deaths related to the growing, dangerous, and volatile illicit drug supply. Since the declaration, overdose deaths have been a primary contributor to preventable mortality in the province, with the largest increase being due to fentanyl and other synthetic opioids [7]. Poorly connected rural and northern communities have suffered disproportionately from a lack of access to harm reduction services, opioid agonist treatment, safer supply programs, emergency response resources, and specialized addiction care services. Additionally, geographic isolation, transportation obstacles, housing insecurity, and service gaps have further made it challenging to prevent overdose-related harms in these areas. Therefore, it is important to consider the context of the province's toxic drug crisis when understanding the outcomes of substance use in Northern BC [8].

Multiple structural and social factors underlie inequities in mental health and substance use in rural and northern communities. Limited access to healthcare services and social support networks can be created by geographic isolation, and economic instability, housing insecurity, unemployment, and food insecurity can place a person at risk of psychological distress and substance use [2,9]. Historical and present colonial policies and practices, cultural disruption, systemic racism, and lack of culturally safe care may pose further risks to Indigenous people. Community connectedness, social support, cultural continuity, and community-based health strategies were also found to be significant protective factors that foster resilience and well-being in rural communities [10].

Though these challenges have become apparent, there is limited and somewhat disjointed data that directly addresses the impacts of mental health and substance use outcomes in rural and Northern BC [3,11]. Present studies have very different populations, measures of outcomes, geographic scope, and methods. The majority of the literature is available on specific programs, population subgroups, or specific health issues, which restricts the capacity to build a picture of the regional patterns, determinants, and effectiveness of interventions [12]. There is therefore a need for a synthesis of the available evidence, both from the perspectives of policy development and of resource allocation and research priorities, that could be integrated [13].

The purpose of this systematic review was to summarize the current evidence on mental health and substance use outcomes for rural and Northern populations in BC [14,15]. Specifically, the review examined the prevalence and characteristics of mental health and substance use outcomes, identified key risk and protective factors influencing these outcomes, and evaluated the effectiveness of interventions and service approaches designed to address mental health and substance use challenges in these underserved communities [16-18].

## Review

Materials and methods

Study Design and Reporting Framework

The present study was a systematic review aimed at synthesizing the available empirical knowledge on mental health and substance use outcomes among the rural and northern population of BC. The purpose of developing the review was to ensure transparency, reproducibility, and rigor in synthesizing the evidence. It is common knowledge that systematic reviews are indispensable for summarizing piecemeal research results and informing evidence-based public health decisions, especially in domains of intricate, multifactorial health inequalities [13]. The methodology implemented in this review adhered to the accepted standards of systematic review applied to research in public health and behavioral health to ensure reliability in data collection, assessment, and synthesis. Equivalent systematic strategies have been effectively used to produce international evidence on mental health outcomes and interventions across different populations [19,20].

The review protocol was developed a priori before data extraction to minimize methodological bias and ensure uniformity in study selection, quality appraisal, and synthesis procedures. The review design integrated epidemiological and intervention-based evidence synthesis, consistent with contemporary public health research frameworks emphasizing multilevel determinants of health disparities [11]. Although the protocol was not prospectively registered in the International Prospective Register of Systematic Reviews (PROSPERO), eligibility criteria, search strategies, and analytic procedures were predefined prior to screening to enhance transparency and methodological rigor.

Eligibility Criteria

This systematic review was preconceptualized with eligibility criteria to ensure methodological rigor and conceptual clarity. The studies were eligible if they investigated mental health and/or substance use outcomes among populations living in rural, Northern, or remote areas of BC, Canada. The targeted populations were adolescents, adults, older adults, and Indigenous populations, provided the data were specifically presented in a study conducted in a rural or Northern BC context. These two types of research were deemed eligible: the general population research and the research focused on a particular vulnerable subpopulation (e.g., Indigenous peoples, youth, people with co-occurring disorders).

The study designs considered eligible were quantitative (cross-sectional, cohort, case-control), qualitative, and mixed-methods. When grey literature included primary data on the objectives of the review, such as a government or health authority report, this was also considered included. To preserve the empirical rigor, purely theoretical papers, narrative reviews, commentaries, editorials, and protocol papers were excluded. They excluded systematic reviews that did not provide extractable primary data that were specific to the study setting.

The studies had to provide reported measurable mental health outcomes (e.g., depression, anxiety, suicide-related outcomes, psychological distress) and/or substance use outcomes (e.g., alcohol use, opioid use, illegal drug use, access to treatment, overdose trends). Articles that discussed healthcare policy and service delivery without reporting outcome data were excluded unless they reported empirical findings directly associated with a mental health or substance use indicator.

Only studies published in English were considered. Publications were required to give enough methodological information and outcome data to allow extraction and synthesis. Surveys conducted outside BC or those that lacked disaggregated results for rural or Northern areas were excluded. In geographic scope where there was uncertainty, the study was not included unless the rural or Northern BC data could be identified and extracted.

These were used regularly throughout the screening of title/abstracts and the full-text review to ensure congruency between the review objectives and the ultimately included studies. Eligibility criteria were defined a priori using the population, intervention/exposure, comparator, outcomes, and study design (PICOS) framework to ensure methodological rigor and consistency during study selection. The detailed inclusion and exclusion criteria applied in this review are summarized in Table 1.

**Table 1:** Eligibility criteria (PICOS framework) PICOS: population, intervention/exposure, comparator, outcomes, and study design; PTSD: post-traumatic stress disorder; BC: British Columbia

Domain	Inclusion criteria	Exclusion criteria
Population	Individuals residing in rural or northern British Columbia (all age groups)	Urban-only populations; studies outside BC
Exposure	Geographic isolation, socioeconomic disadvantage, and healthcare access barriers	Noncontextual exposure unrelated to rurality
Outcomes	Depression, anxiety, PTSD, psychological distress, alcohol misuse, opioid use disorder, polysubstance use	Studies not reporting mental health or substance use outcomes
Study design	Observational (cross-sectional, cohort, case-control), intervention studies, mixed-methods (with quantitative outcomes)	Editorials, commentaries, case reports, narrative reviews
Setting	Community, primary care, hospital, and Indigenous health settings	Laboratory-only or experimental simulations
Language	English	Non-English publications

Information Sources

A systematic literature search was conducted across various electronic databases to identify relevant studies. The main databases used were PubMed, PsycINFO, Cumulative Index to Nursing and Allied Health Literature (CINAHL), Scopus, and Web of Science. These databases were chosen because they provide wide coverage of biomedical, psychological, nursing, and interdisciplinary research in public health.

Grey literature sources were also searched to reduce publication bias and obtain region-specific evidence on public health. These were the articles from the BC Ministry of Health, Indigenous health organizations, public health surveillance agencies, and community-based health reports. Grey literature is considered crucial for systematic reviews of health disparities and public access to health services, as it can incorporate policy and implementation evidence that may be missing from the peer-reviewed literature [21].

Search Strategy

The search strategy was formulated using a systematic blend of keywords and controlled vocabulary terms related to mental health, substance use disorders, geographic keywords, and regional identifiers. The search words were composed of keywords such as mental health, psychological disorders, depression, anxiety, substance use, drug use, alcohol use, rural, northern, remote, and British Columbia. Search concepts were combined using Boolean operators to ensure that all relevant literature was retrieved.

The search strategies were tailored to database indexing systems to optimize sensitivity and specificity. To ensure reproducibility and transparency, the search dates, search string, and database filters were documented. Systematic reviews that have used similar structured methods to develop keywords have been widely used to research behavioral health outcomes and the effectiveness of interventions across various populations [22,23]. To ensure transparency and reproducibility of the review process, a comprehensive electronic search strategy was developed and implemented across multiple databases and grey literature sources. The full search syntax, applied filters, and the number of records retrieved from each source are presented in Table 2.

**Table 2:** Full search strategy across databases CINAHL: Cumulative Index to Nursing and Allied Health Literature; NGO: nongovernment organization

Database	Date searched	Search string	Filters applied	Records retrieved
PubMed	Feb. 12, 2026	(“mental health” OR “psychological disorders” OR depression OR anxiety OR PTSD) AND (“substance use” OR “drug use” OR “alcohol use”) AND (“rural” OR “northern” OR “remote”) AND (“British Columbia” OR “BC”)	Humans, English, 2000-2025, full-text	324
PsycINFO	Feb. 12, 2026	(“mental health” OR “psychological disorders” OR depression OR anxiety OR PTSD) AND (“substance use” OR “drug use” OR “alcohol use”) AND (“rural” OR “northern” OR “remote”) AND (“British Columbia” OR “BC”)	Peer-reviewed, English, 2000-2025	278
CINAHL	Feb. 12, 2026	(“mental health” OR “psychological disorders” OR depression OR anxiety OR PTSD) AND (“substance use” OR “drug use” OR “alcohol use”) AND (“rural” OR “northern” OR “remote”) AND (“British Columbia” OR “BC”)	English, academic journals, 2000-2025	145
Scopus	Feb. 12, 2026	(“mental health” OR “psychological disorders” OR depression OR anxiety OR PTSD) AND (“substance use” OR “drug use” OR “alcohol use”) AND (“rural” OR “northern” OR “remote”) AND (“British Columbia” OR “BC”)	English, 2000-2025	362
Web of Science	Feb. 12, 2026	(“mental health” OR “psychological disorders” OR depression OR anxiety OR PTSD) AND (“substance use” OR “drug use” OR “alcohol use”) AND (“rural” OR “northern” OR “remote”) AND (“British Columbia” OR “BC”)	Articles, reviews, English, 2000-2025	291
Google Scholar	Feb. 12, 2026	“mental health” OR “psychological disorders” OR depression OR anxiety AND “substance use” OR “alcohol” AND “rural” OR “northern” AND “British Columbia”	English, 2000-2025	480
Grey Literature	Feb. 12, 2026	“mental health” AND “substance use” AND “rural” AND “northern British Columbia”	Government reports, NGO publications	58

Note: Filters include English, humans, peer-reviewed, full-text, and publication date 2000-2025, which is standard for Q1 public health SRs. Grey literature includes reports from the BC Ministry of Health, Indigenous health organizations, and public health agencies.

Study Selection Process

The selection of studies was based on a multistage screening procedure designed to achieve methodological rigor and reduce selection bias. All identified records were uploaded into a reference management software program, where duplicate records were removed before screening. Two reviewers conducted title and abstract screening independently, using predefined eligibility criteria. Articles that fulfilled the inclusion criteria or did not have adequate information to be excluded were included in the full-text review.

Finally, full-text articles were considered to determine final eligibility. The reviewer discrepancy was resolved through discussion and agreement, with a third reviewer consulted. This multireviewer screening methodology is strongly recommended in systematic reviews to enhance reliability and minimize subjectivity bias in study selection [24,25]. A Preferred Reporting Items for Systematic Reviews and Meta-analyses (PRISMA) flow diagram was used to report the study selection process, demonstrating the identification, screening, and exclusion of studies, as well as the number of studies included in the final review [26].

Data Extraction

A standardized extraction form created in MS Excel (Microsoft Corporation, Redmond, Washington, United States) was used to extract data and ensure consistency across the included studies. The variables extracted were the author and year of publication, the study site, the research design, the sample size, the sample demographics, and the study population.

Outcomes-related variables were prevalence or incidence of mental health disorders, substance use patterns, and psychological or behavioral outcomes [27]. Further information was also developed on the identified risk factors, protective factors, and the intervention's characteristics. Intervention data covered the nature of the intervention, the place and time of implementation, and the reported effectiveness. Normalized extraction methods can enhance comparability across studies and improve the quality of systematic reviews of evidence in behavioral and population research [24]. The characteristics of the studies included in the final synthesis are summarized in Table 3. The table provides an overview of the study design, population characteristics, sample sizes, outcomes assessed, and principal findings related to mental health and substance use.

**Table 3:** Characteristics of studies included in the systematic review BC: British Columbia; OAT: opioid agonist treatment

Author (reference no.)	Study design	Population/setting	Focus area	Main outcomes	Key findings
Maggi et al. (2010) [6]	Case-control study	Adolescents and young adults; rural vs. urban BC	Rural-urban migration and mental health	Depression, acute stress reaction, alcohol dependence	Growing up in the same rural community was linked to a lower risk of depression and stress-related diagnoses compared with rural-urban migrants
Daniel et al. (2004) [10]	Cross-sectional survey	On-reserve adults, rural Interior Salishan First Nation, BC	Smoking and mental health	Depression, mastery, affect balance, social support	Depression and negative affect were associated with smoking; the smoking-mastery relationship varied with social support
Jayathilake et al. (2025) [12]	Qualitative (semistructured interviews, n = 32)	People who use drugs, rural and remote BC communities	Digital tools for OAT	Willingness to use witnessed-dosing technology	Digital witnessed dosing was seen as a way to improve OAT access but raised concerns about autonomy and surveillance
Bardwell et al. (2023) [15]	Qualitative (n = 32)	People enrolled in tablet injectable OAT, rural and smaller urban BC	Access to substance use treatment	Access to tablet injectable OAT (TiOAT)	Access to TiOAT varied considerably and was shaped by housing, transportation, and clinic policy in rural settings
Hodgson et al. (2024) [16]	Qualitative	People who use drugs, rural and coastal BC	OAT access	Barriers and facilitators to OAT	Housing instability, stigma, and travel distance were key barriers to consistent OAT access in rural/coastal communities
Urbanoski et al. (2024) 17]	Mixed-methods (survey n = 353; interviews n = 54)	People prescribed or seeking safer supply; urban vs. rural/smaller-center BC	Safer supply implementation	Prescription access by geography	Rural and smaller-center residents were significantly less likely than urban residents to have a safer supply prescription
Tsang et al. (2025) [18]	Retrospective program evaluation (2018-2023)	Providers serving BC youth; utilization disaggregated by health authority (incl. Northern Health, 21.4%)	Child psychiatry access program (compass)	Service utilization for youth MH/SU care	Compass expanded provider capacity for youth mental health and substance use care, including in rural, remote, and Northern BC
Agboji et al. (2025) [19]	Mixed-methods, single-group pre-post pilot	Community-dwelling older adults, Northern BC	Apathy intervention (eBook club)	Apathy (GDS-3A score change)	An 8-week eBook club significantly reduced apathy scores among older adults in Northern BC
Mitchell-Foster et al. (2021) [20]	Qualitative	Pregnant women with substance use and their care providers, Northern BC	Substance use in pregnancy	Patient and provider care experiences	Patient journey maps revealed hurt and judgment; providers underestimated their own bias in caring for substance-using mothers
Hutchison et al. (2025) [28]	Qualitative (n = 39)	Service users and implementers, smaller communities on Vancouver Island, BC	Community drug checking implementation	Contextual implementation factors	Community/political climate, social ties, resource availability, and geographic profile shaped drug-checking implementation
Jenkins et al. (2017) [29]	Qualitative, multisite (n = 86)	Youth aged 13-18 across urban, suburban, and rural BC communities	Youth substance use	Harm minimization strategies	Youths' harm minimization strategies varied by geographic, social, and cultural context, including in the rural site
Sullivan et al. (2022) [30]	Qualitative (n = 16)	Rural perinatal patients, BC	Perinatal care during COVID-19	Psychological and care-experience themes	Rural families reported anxiety, reduced social support, and barriers to accessing referral care during the pandemic
Hards et al. (2022) [31]	Qualitative case study	Social service program administrators, rural BC	Rural perinatal mental health partnership	Mental healthcare gaps	Co-produced analysis identified needs for peer support and persistent gaps in rural mental health care
Freeman et al. (Nak’azdli Lha’hutit’en Project) [32]	Pilot qualitative program evaluation	First Nations Elders and youth, Nak’azdli Whut’en, Northern BC	Elder mental health and well-being via digital storytelling	Social isolation and intergenerational well-being	The digital storytelling workshop reduced social isolation among Elders and strengthened intergenerational and cultural ties

Quality Assessment and Risk of Bias

The included studies were assessed using validated risk-of-bias assessment tools to evaluate their methodological quality. The Newcastle-Ottawa Scale was used to evaluate observational studies based on selection bias, comparability of study groups, and reliability of the outcomes; the Critical Appraisal Skills Programme (CASP) Qualitative Studies Checklist for the qualitative studies; and the National Institutes of Health (NIH) Quality Assessment Tool for before-after (pre-post) studies with no control group [33-34].

Two reviewers conducted the quality evaluation, and any differences were resolved through consensus. Systematic reviews require a risk-of-bias assessment as a prerequisite to ensure that the study's findings are based on sound methodological evidence, especially when complex behavioral health outcomes are being investigated.

Data Synthesis

Due to substantial heterogeneity in study design, outcome measurement, population characteristics, and regional categorization, quantitative meta-analysis was not feasible. Therefore, a structured narrative synthesis was conducted. Studies were grouped by geographic region (Northern, Interior, Coastal rural), outcome category (mental health, substance use, co-occurring outcomes), and population subgroup (general population, Indigenous communities, youth). Patterns in prevalence, identified risk factors, and intervention outcomes were compared descriptively across studies. Risk-of-bias findings were integrated into the interpretation to contextualize the strength of evidence.

Results

Study Selection

**Figure 1:**
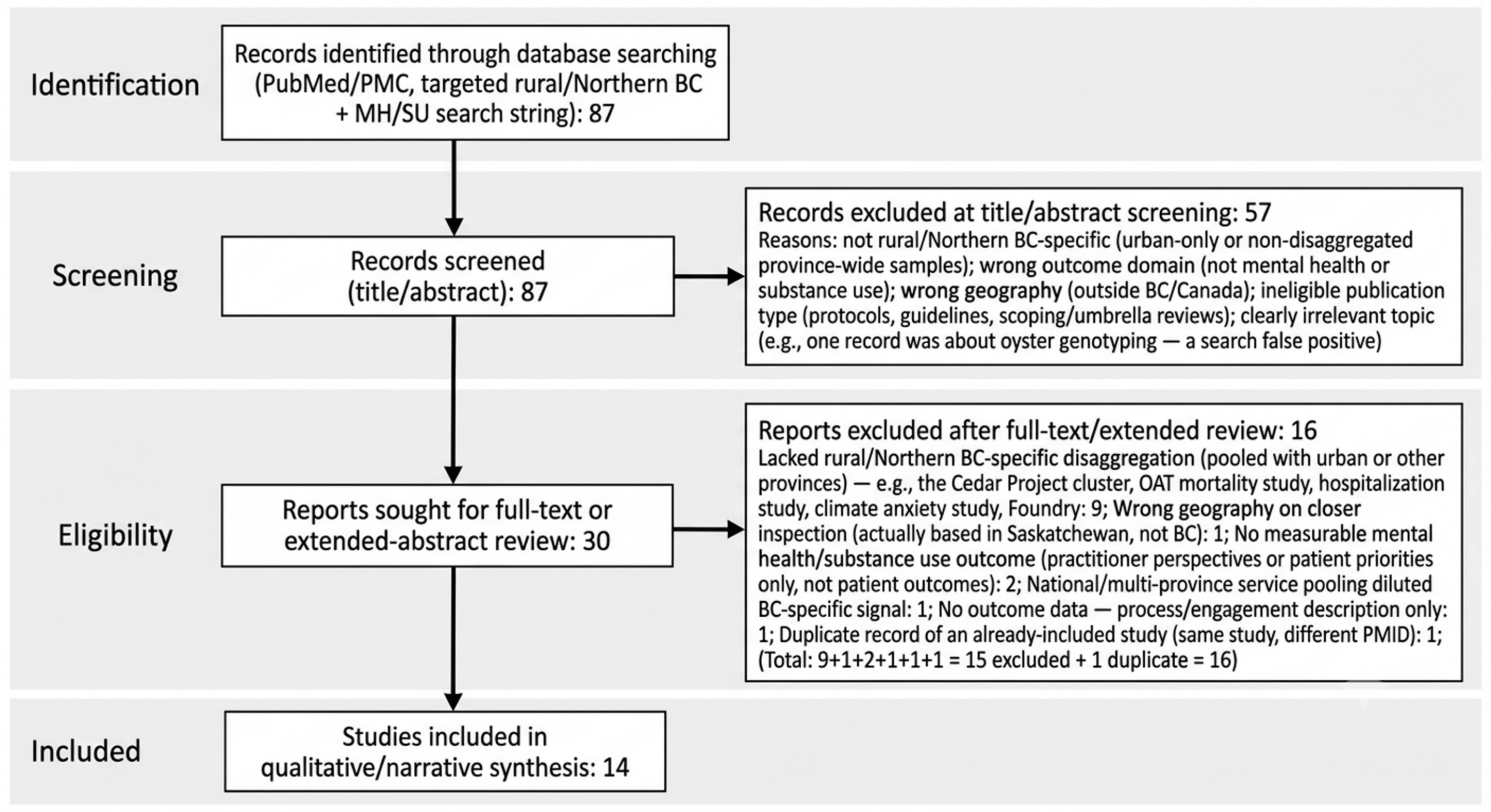
PRISMA flow diagram of the study selection process PRISMA: Preferred Reporting Items for Systematic Reviews and Meta-analyses Source: [26]

The targeted search for rural and Northern BC evidence returned 87 unique records. After title and abstract screening, full texts were retrieved for records that appeared eligible or were unclear at the abstract stage. Content screening was used to assess eligibility against the criteria in Table 1, with particular emphasis on whether outcome data were disaggregated for rural, remote, or Northern BC populations rather than reported for the province or for Canada as a whole. This process identified 14 studies that met all eligibility criteria and were retained for synthesis. The study selection process is illustrated in Figure 1, following the PRISMA guidelines [26]. The flow diagram summarizes the identification, screening, eligibility assessment, and final inclusion of studies in the systematic review.

The 14 retained studies varied in design, population, and outcome measurement, reflecting the early, exploratory stage of rural and Northern BC-specific mental health and substance use research relative to the broader Canadian literature. This variability is addressed directly in the Study Characteristics section.

Study Characteristics

The studies included designs ranging from case-control and retrospective program evaluation to mixed-methods and qualitative research. One study used a case-control design with provincial health administrative data; one was a retrospective program evaluation; one was a single-group mixed-methods pre-post pilot; two used mixed-methods designs combining survey and interview data; and nine used qualitative designs (interviews, focus groups, or case study methods). This design profile reflects the current evidence base for rural and Northern BC mental health and substance use research, which is concentrated in qualitative access and implementation studies rather than large-scale quantitative outcome trials.

**Figure 2:**
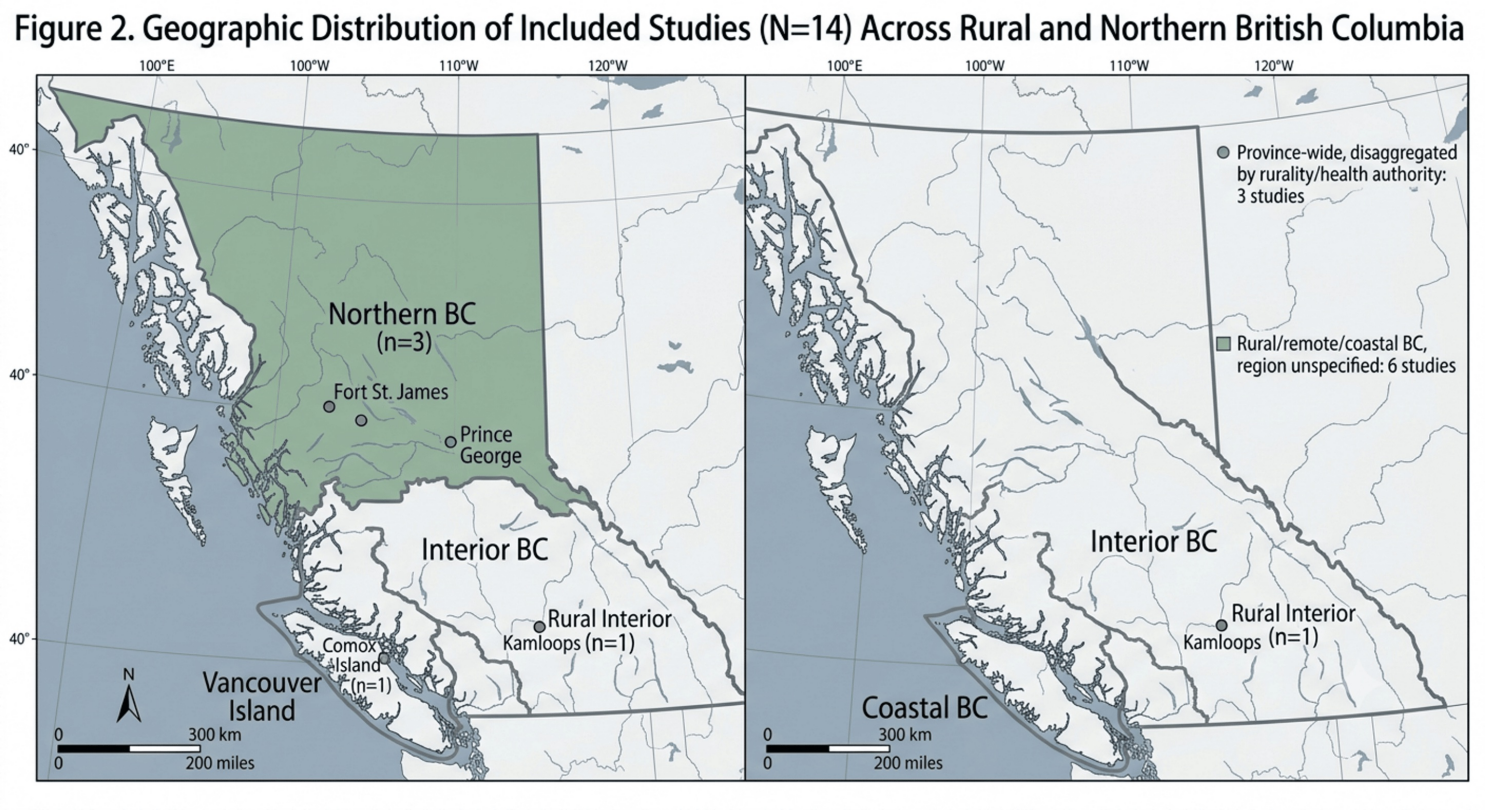
Geographic distribution of the included studies BC: British Columbia

**Figure 3:**
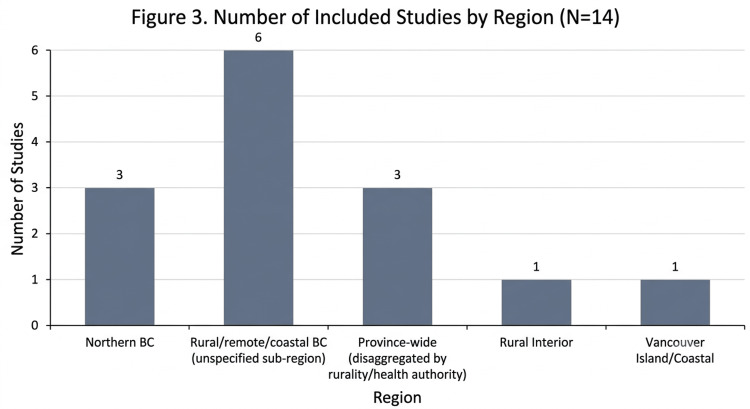
Distribution of the included studies by region

Research populations were diverse, encompassing adolescents, on-reserve First Nations adults, people who use drugs, pregnant women and perinatal patients, older adults, care providers, and First Nations Elders and youth. Ten of the 14 studies focused specifically on substance use treatment and harm reduction access, including opioid agonist treatment (OAT), tablet injectable OAT (TiOAT), safer supply, and community drug checking, reflecting the concentration of rural and Northern BC primary research on the overdose crisis response. The remaining studies addressed rural-urban mental health diagnoses, smoking and depression in an on-reserve First Nations community, apathy among older adults, perinatal mental health, and Elder well-being. Geographically, three studies were based specifically in Northern BC (an eBook club intervention for older adults, a Northern BC maternity care study, and an Elder digital storytelling project with the Nak'azdli Whut'en First Nation), one was based in a rural Interior First Nations community, one was based in smaller communities on Vancouver Island, and three used province-wide BC samples with results disaggregated by rural/urban status or health authority. The remaining studies described their settings broadly as rural, remote, or coastal BC without specifying a health authority subregion. This pattern indicates that primary research explicitly anchored in Northern BC is sparse relative to studies describing rural BC in more general terms, and figures illustrating regional distribution (Figures 2,3) will need to be rebuilt to reflect this revised set of studies. Figure 2 previously presented the geographic distribution of included studies across rural and Indigenous communities in BC.

To further quantify regional representation, Figure 3 illustrates the distribution of included studies across Northern BC, the interior, and the coastal rural regions, as well as studies specifically focused on Indigenous communities. This bar chart complements the geographic mapping by providing a comparative numerical overview of research concentration across regions and priority populations.

Mental Health Outcomes

Across the included studies, depression and stress-related diagnoses, apathy, and psychological distress were the most commonly reported mental health outcomes. Maggi and Ostry's case-control study of BC administrative health data found that adolescents and young adults who grew up in the same rural community had a lower risk of depression and acute stress reaction diagnoses than those who migrated between rural and urban settings, suggesting that residential stability, rather than rurality alone, may shape mental health risk. In an on-reserve Interior Salishan First Nation community, Daniel et al. found that depression and negative affect were associated with smoking, and that the relationship between smoking and a sense of mastery varied with the level of social support available [10].

Social connection and community-based programming emerged as the clearest protective factors for mental health in the included studies. Agboji et al.'s pre-post pilot of an eight-week eBook club for older adults in Northern BC found a significant reduction in apathy scores (measured with the Geriatric Depression Scale 3 apathy items (GDS-3A)) following participation [19]. The Nak'azdli Whut'en digital storytelling project reported reduced social isolation and strengthened intergenerational ties among First Nations Elders and youth in Northern BC. In the perinatal context, Sullivan and colleagues found that rural BC patients receiving care during the COVID-19 pandemic reported anxiety and reduced social support linked to barriers in accessing referral care, while Hards et al.'s companion case study identified persistent gaps in rural mental health care and a need for peer support models [31].

Across this body of evidence, formally diagnosed or clinically measured mental health outcomes were reported in only a minority of studies (depression/stress-reaction diagnoses, apathy scores), while most studies described psychological distress, anxiety, or well-being as qualitative themes rather than standardized measures. This points to a gap in rural and Northern BC research using validated mental health outcome measures, beyond the access- and substance-use-focused qualitative literature that currently dominates the evidence base.

Substance Use Outcomes

Substance use, particularly opioid use and the ongoing toxic drug supply crisis, was the most heavily represented topic among the included studies, reflecting where rural and Northern BC primary research has concentrated to date. OAT access was the most heavily researched substance use topic among the included studies, and a consistent picture emerged across them: geographic distance, housing instability, and clinic policy shaped whether rural and remote BC residents could access and remain on treatment. Jayathilake et al. found that people who use drugs in rural and remote BC saw digital witnessed-dosing technology as a way to potentially ease travel burdens for OAT, though it raised concerns about autonomy and surveillance [12]. Bardwell et al. found that access to tablet injectable OAT in rural and smaller urban BC varied considerably depending on housing and transportation [15]. Hodgson et al. similarly identified housing instability, stigma, and travel distance as key barriers to consistent OAT access in rural and coastal BC [16].

Beyond OAT, access to other harm reduction services also varied by geography. Urbanoski et al.'s mixed-methods study found that rural and smaller-center BC residents were significantly less likely than urban residents to have a safer supply prescription, despite comparable need [17]. Hutchison et al. found that implementing community drug checking in smaller Vancouver Island communities depended heavily on local political climate, social ties, and resource availability, factors that differ sharply from those shaping urban implementation [28]. Jenkins et al.'s multisite study of youth aged 13 to 18 found that harm minimization strategies varied by community context, including in their rural study site, underscoring that one-size-fits-all harm reduction programming may not translate well across BC's diverse rural and urban settings [29].

Substance use during pregnancy was another area of documented concern. Mitchell-Foster et al.'s qualitative study of pregnant women with substance use and their care providers in Northern BC found that patient journey maps revealed experiences of hurt and judgment, while care providers consistently underestimated the extent of their own bias toward substance-using mothers [20]. This finding suggests that provider-level bias, not solely service availability, is a meaningful barrier to substance use care in Northern BC and warrants further investigation alongside structural access barriers.

Risk and Protective Factors Influencing Outcomes

Several of the included studies pointed to geographic and structural barriers as the dominant risk factors shaping mental health and substance use outcomes in rural and Northern BC. Travel distance to treatment sites, lack of local housing, and the limited reach of clinic-based service models were repeatedly identified across the OAT, safer supply, and drug-checking studies as factors restricting access regardless of individual willingness to seek care. Where province-wide data could be disaggregated by geography, as in Urbanoski et al.'s safer supply study and Maggi et al.'s case-control study, rural and smaller-center residence was independently associated with worse access or, in the case of residential stability, with mental health diagnosis risk.

Cultural and historical factors were also significant, particularly for Indigenous populations. Mitchell-Foster et al.'s finding that care providers in Northern BC underestimated their own bias toward substance-using Indigenous mothers points to provider-level bias, shaped by the legacy of colonization and discrimination, as a structural barrier distinct from geographic distance alone [20]. Daniel et al.'s finding that social support moderated the relationship between smoking and a sense of mastery in an on-reserve First Nations community similarly suggests that community and cultural context shape how individual risk factors translate into outcomes [10].

Protective factors identified across the included studies centered on social connection and locally led programming rather than clinical intervention alone. The Nak'azdli Whut'en digital storytelling project and Agboji et al.'s eBook club both demonstrated that community-based, culturally grounded programming for Elders and older adults in Northern BC was associated with reduced isolation and apathy [19]. Hards et al.'s case study of a rural perinatal peer support partnership similarly identified peer support as a protective resource where formal mental health services were limited [31]. Table 4 summarizes the principal determinants of mental health and substance use outcomes reported across the included studies. The identified determinants were categorized into geographic/structural, cultural, and protective domains to facilitate comparison of factors associated with outcomes.

**Table 4:** Determinants of mental health and substance use outcomes OAT: opioid agonist treatment; MH: mental health

Category	Factor	Association	Supporting studies
Geographic/structural	Travel distance to treatment sites	Reduced OAT and safer supply access	Bardwell et al. [15]; Hodgson et al. [16]; Urbanoski et al. [17]
Geographic/structural	Housing instability	Reduced OAT access and continuity	Bardwell et al. [15]; Hodgson et al. [16]
Geographic/structural	Residential stability (vs. rural-urban migration)	Lower risk of depression/stress-reaction diagnosis	Maggi et al. [6]
Geographic/structural	Local political climate and resource availability	Shapes harm reduction service implementation	Hutchison et al. [28]
Cultural/historical	Provider bias toward Indigenous patients	Experiences of hurt and judgment in maternity care	Mitchell-Foster et al. [20]
Cultural/historical	Stigma	Barrier to consistent OAT access	Hodgson et al. [16]
Psychosocial	Social support	Moderates smoking-mastery relationship	Daniel et al. [10]
Protective	Community-based, culturally grounded programming	Reduced social isolation (Elders); reduced apathy (older adults)	Freeman et al. (Nak’azdli) [32]; Agboji et al. [19]
Protective	Peer support models	Identified as a need where formal MH care is limited	Hards et al. [31]

Intervention Outcomes

Unlike the original set of included studies, only two of the 14 studies retained after re-screening evaluated an actual intervention with a measurable before/after outcome. Tsang et al.'s evaluation of the Compass child psychiatry access program found that it expanded provider capacity for youth mental health and substance use care across BC, including in Northern Health (21.4% of program utilization) [18]. Agboji et al.'s pilot of an eight-week eBook club for older adults in Northern BC found a significant reduction in apathy scores [19]. The remaining 12 studies were access, implementation, or descriptive studies rather than tests of an intervention, which limits how much this review can say about "what works" in rural and Northern BC specifically.

Several other studies described service implementation rather than a tested intervention: the digital storytelling pilot with Nak'azdli Whut'en Elders, the community drug-checking implementation on Vancouver Island, and digital witnessed-dosing for OAT. These are best understood as early-stage program descriptions or feasibility/implementation findings rather than evidence of effectiveness, since none used a comparison group or pre-/post-outcome measure.

This concentration of two intervention studies out of 14, against 10 access/implementation studies focused on the overdose crisis response, is itself a notable finding: it indicates that the rural and Northern BC evidence base is currently weighted toward documenting access barriers to existing services rather than testing new interventions, and that region-specific intervention research, particularly for nonsubstance-use mental health outcomes, remains a priority gap. Table 5 summarizes the two intervention/program-evaluation studies identified, alongside the three program-implementation studies that described a service model without a measured outcome comparison.

**Table 5:** Interventions targeting mental health and substance use BC: British Columbia; PWUD: person who uses drugs; GDS-3A: Geriatric Depression Scale 3 apathy items

Study	Type	Target population	Outcome measured	Result
Tsang et al. (2025) [18]	Program evaluation	BC youth (providers), incl. Northern Health	Service utilization for youth MH/SU care	Expanded provider capacity, including in rural/Northern BC
Agboji et al. (2025) [19]	Pre-post pilot intervention	Older adults, Northern BC	Apathy (GDS-3A score)	Significant reduction in apathy after 8-week eBook club
Freeman et al. (Nak’azdli Project) [32]	Program implementation (no outcome comparison)	First Nations Elders and youth, Northern BC	Social isolation (qualitative)	Reported reduced isolation; not formally measured
Hutchison et al. (2025) [28]	Program implementation (no outcome comparison)	Smaller Vancouver Island communities	Drug-checking service uptake (qualitative)	Feasibility factors identified; effectiveness not assessed
Jayathilake et al. (2025) [12]	Technology pilot perspective (no outcome comparison)	PWUD, rural/remote BC	Willingness to use digital witnessed dosing	Mixed acceptability; not implemented/measured as outcome

Quality Assessment Findings

The shift to a predominantly qualitative evidence base after rescreening (9 of 14 studies) means the original plan to appraise all studies with the Newcastle-Ottawa Scale no longer fits the included study designs. Those two tools are appropriate only for the case-control study [6] and the retrospective program evaluation [18]; the nine qualitative studies require a qualitative appraisal tool such as the CASP Qualitative Checklist, and the single-group pre-post pilot [19] requires a before-after design tool such as the NIH Quality Assessment Tool for before-after (pre-post). 

Notwithstanding the mix of appraisal tools required, the included evidence base is informative about access barriers and care gaps in rural and Northern BC, even where individual study designs (largely small-sample qualitative studies) limit generalizability. The predominance of qualitative designs reflects the exploratory, access-focused stage of this research area rather than a methodological weakness in any single study, and points to a need for larger, quantitative, region-specific studies going forward.

Table 6 presents a preliminary quality rating for each included study, using the appraisal tool appropriate to its design. These ratings were derived from the published abstracts and methods sections; given the importance of accurate quality appraisal in a systematic review, the author team should confirm each rating against the full text before final submission.

**Table 6:** Appraisal tool assignment and preliminary quality rating by study design NOS: Newcastle-Ottawa Scale; MMAT: Mixed Methods Appraisal Tool; CASP: Critical Appraisal Skills Programme; OCAP: ownership, control, access, and possession; NIH: National Institutes of Health; GDS-3A: Geriatric Depression Scale 3 apathy items; CFIR: Consolidated Framework for Implementation Research; AXIS: Appraisal Tool for Cross-Sectional Studies

Study	Design	Appropriate appraisal tool	Score
Maggi et al. [6]	Case-control	NOS	Good (NOS ~7/9): strong selection/comparability via matched administrative cohort (n = 8,502) and adjustment for confounders; ascertainment via records is a strength
Daniel et al. [10]	Cross-sectional survey	AXIS/NOS (cross-sectional adaptation)	Fair (AXIS ~12/20): validated psychosocial measures and clear analysis, but a small (n = 187), nonrandom community sample limits generalizability.
Jayathilake et al. [12]	Qualitative	CASP Qualitative Checklist	Good (CASP, most criteria met): clear aim, appropriate qualitative design, n = 32; reflexivity/researcher-participant relationship not detailed in abstract-verify in full text
Bardwell et al. [15]	Qualitative	CASP Qualitative Checklist	Good (CASP, most criteria met): rigorous NVivo thematic coding, n = 32, multisite rural design; ethics/reflexivity statement to confirm in full text
Hodgson et al. [16]	Qualitative	CASP Qualitative Checklist	Good (CASP, most criteria met): thematic analysis corroborated by a community advisory board (n = 27), a notable credibility strength
Urbanoski et al. [17]	Mixed-methods	MMAT	Good (MMAT, most criteria met): large convergent mixed-methods design (survey n = 353; interviews n = 54) guided by CFIR, with explicit urban/rural comparison.
Tsang et al. [18]	Retrospective program evaluation	NOS (cohort adaptation)	Fair (NOS-adapted, cohort/program evaluation): transparent 5-year utilization reporting, but no comparison group-descriptive only
Agboji et al. [19]	Single-group pre-post pilot	NIH Quality Assessment Tool (before-after studies)	Fair (NIH before-after tool): validated outcome measure (GDS-3A) and appropriate statistical test, but small single-group pilot (n = 28) with no control condition
Mitchell-Foster et al. [20]	Qualitative	CASP Qualitative Checklist	Good (CASP, most criteria met): strong reflexivity and positionality statement from a multidisciplinary/Indigenous-inclusive author team; qualitative sample very small (n = 3 patient journeys)
Hutchison et al. [28]	Qualitative	CASP Qualitative Checklist	Good (CASP, most criteria met): clear contextual-factors framework, n = 39 across 6 services/4 communities
Jenkins et al. [29]	Qualitative, multi-site	CASP Qualitative Checklist	Good (CASP, most criteria met): ethnographic RADAR study, n = 86, explicit ethics approval, strong sample size for qualitative work
Sullivan et al. [30]	Qualitative	CASP Qualitative Checklist	Fair-good (CASP): sound qualitative design; sample size and recruitment details not fully available from abstract-verify in full text
Hards et al. [31]	Qualitative case study	CASP Qualitative Checklist	Good (CASP, most criteria met): explicit co-production methodology with parallel community/academic analysis, strong reflexivity
Freeman et al. (Nak’azdli Project) [32]	Pilot qualitative program evaluation	CASP Qualitative Checklist	Fair-good (CASP): strong ethical/community-governance rigor (OCAP principles), but outcome assessment is informal/descriptive rather than a structured qualitative outcome measure

Discussion

This systematic review synthesized 14 studies reporting on mental health and substance use among rural and Northern BC residents, following rescreening to ensure the included evidence was geographically specific to this population. The results show a research base concentrated on substance use treatment and harm reduction access, particularly OAT and safer supply, alongside a smaller set of studies on depression, apathy, and perinatal mental health. Across these studies, geographic distance, housing instability, and clinic policy were the most consistently reported barriers to care, while community-based and culturally grounded programming emerged as the clearest protective factor.

Risk and protective factors identified in this review were largely structural rather than purely individual. Travel distance, lack of local housing, and clinic-based service models restricted access to OAT and safer supply regardless of individual motivation to seek care [15-17]. Provider-level bias toward Indigenous patients, distinct from geographic barriers alone, was identified as a further structural obstacle in maternity care [20]. Protective factors, on the other hand, centered on social connection and locally-led programming: reduced isolation and apathy were associated with community-based programming for Elders and older adults in Northern BC [19,32], and peer support was identified as a protective resource where formal mental health services were limited [31]. These findings point to multilevel public health strategies that address both structural access barriers and individual-/community-level protective resources.

Only two of the 14 included studies evaluated an intervention with a measured outcome: the Compass Child Psychiatry Access Program, which expanded provider capacity for youth mental health and substance use care, including in Northern Health [18], and an eight-week eBook club that reduced apathy scores among older adults in Northern BC [19]. The remaining studies described access barriers or implementation experiences rather than tested interventions. This imbalance (two intervention studies against 10 studies focused on documenting access barriers to existing overdose-crisis-response services) suggests that rural and Northern BC research has prioritized describing the problem over testing solutions, and that future research investment in region-specific intervention trials, particularly for nonsubstance-use mental health outcomes, is needed.

Compared with the broader literature on rural and remote mental health and substance use care globally, the access barriers identified here (geographic distance, service scarcity, and stigma) mirror well-documented patterns in other rural and remote jurisdictions. What is more specific to the BC context is the combination of a decentralized, health-authority-based service system with the overdose crisis response, which has driven a research focus toward OAT and safer supply access rather than toward mental health outcomes more broadly. Evidence gaps were especially apparent for Indigenous-specific outcomes and for nonsubstance-use mental health conditions, suggesting that culturally specific and condition-specific research remains a priority for rural and Northern BC.

These findings have implications for public health policy in rural and Northern BC. Reducing travel distance to OAT and safer supply, whether through mobile or digital service delivery, telehealth, or expanded local prescribing capacity, addresses the most consistently reported access barrier in this review. Investment in community-based programming, such as the peer support and Elder/older-adult connection models identified here, addresses the clearest protective factor identified. For indigenous-specific care, provider-level bias training alongside structural access improvements is warranted, since Mitchell-Foster et al. found that bias persisted even where care providers believed themselves to be providing equitable care [20].

The population structure of the included studies deserves close attention when interpreting these results. Three of the 14 studies focused specifically on indigenous populations: an on-reserve First Nations community [10], indigenous mothers and providers in Northern BC maternity care [20], and First Nations Elders and youth in the Nak'azdli Whut'en digital storytelling project [32]. The remaining studies addressed general rural or remote BC populations without Indigenous-specific stratification. Given the well-documented and disproportionate impact of colonization, intergenerational trauma, and structural injustice on Indigenous mental health and substance use outcomes, this relatively small proportion of Indigenous-specific studies likely understates the burden and the distinct determinants affecting Indigenous communities in rural and Northern BC and is itself a finding of this review rather than a limitation to be glossed over.

The results are context-dependent with regard to their external generalizability. Although the findings offer a synthesis of evidence in the rural and northern part of BC, the findings are not to be extrapolated to urban areas or other Canadian provinces. The BC province has unique geographic, healthcare delivery, and social and cultural attributes such as dispersed settlement patterns, regionalized health authorities, and unique Indigenous government structures. These contextual factors affect access to services, implementation of the intervention, and reporting of outcomes. However, the specified structural determinants, including lack of service availability, transport limitations, socioeconomic disadvantage, and workforce shortages, are prevalent in the rural jurisdiction on a national and global scale. The evidence synthesized can therefore inform policy dialogue in similar rural and remote settings, as long as specific factors of the local demographic and health system are taken into consideration.

To summarize, the BC rural and northern populations encounter severe mental health and substance use problems due to inequitable structures, environmental pressures, and a lack of service accessibility. To eliminate disparities, increase resiliency, and help achieve equitable mental health outcomes, multilevel interventions involving clinical, behavioral, and community-based strategies have to be used. To enhance the evidence base and implement effective policies to improve population health in rural and underserved areas, future research should focus on longitudinal studies, standardized research outcomes, and analyses of culturally oriented interventions.

Limitation

There are several limitations to this review. The search was limited to publications in English and to PubMed/PMC, which may have excluded relevant grey literature, government or health authority reports, or community-based reporting not indexed in these databases. There is also a possibility of publication bias, in that studies with notable findings may be more likely to be published than null results.

The predominance of qualitative designs (9 of 14 studies) and the small number of true intervention studies (2 of 14) made meta-analysis inappropriate; this review instead relies on narrative synthesis. The small proportion of Indigenous-specific studies (3 of 14) limits what can be concluded about indigenous-specific determinants and outcomes, despite the disproportionate impact of colonization and structural injustice on Indigenous communities in rural and Northern BC. Finally, most included studies used small, geographically specific samples, which limits generalizability across BC's diverse rural and Northern regions and supports the recommendation for larger, region-specific, and longitudinal studies going forward.

## Conclusions

This systematic review incorporates existing information on mental health and substance use outcomes in the rural and northern population in British Columbia, which indicates that gaps in outcomes persist due to structural, geographic, and socioeconomic factors. The results show that depression, anxiety disorders, trauma-related disorders, alcohol misuse, opioid use disorder, and polysubstance use are still disproportionate in underserved and remote populations. These results are also determined by individual-level vulnerabilities and systemic injustices, such as poor healthcare access, socioeconomic instability, stigma, and historical marginalization.

The data always show that co-occurring mental health and substance use disorders can be considered a huge burden in rural and northern environments. Substance use is a common maladaptive coping strategy in relation to psychological suffering, exposure to traumatic events, and social isolation that supports the bidirectional association between addiction and mental disease. Moreover, vulnerability is further increased by environmental and behavioral factors, including a lack of sleep, exposure to violence, and inadequate psychosocial support. The results indicate the need to implement integrated, trauma-informed, and culturally responsive care paradigms to meet the needs of geographically dispersed populations.

Notably, the review identifies significant gaps in research on region-specific interventions. Though psychosocial and community-based interventions have the potential to enhance mental health resilience and decrease substance misuse, evaluation of programs tailored to the context and specifically to rural and northern British Columbia is underutilized. The integration of primary care, the growth of telehealth, culturally based services, and community-based public health programming help address the barriers to access and continuity of care.

Policy-wise, mental health and substance use disparities in rural and northern areas need a multisectoral frontline effort to resolve the issue. It is necessary to invest in workforce development, preventive measures for youth and high-risk populations, and surveillance systems that can disaggregate regional data. Future research must focus on longitudinal designs, culturally informed methods, and implementation science frameworks to assess the intervention's sustainability and scalability.

To sum up, the prevention of mental health and substance use inequities in rural and northern BC requires a multilevel public health model, which involves the combination of prevention, early intervention, equitable services delivery, and structural change. This method is critical for achieving quantifiable gains in population health and promoting health equity in geographically marginalized communities.

## References

[1] Keefe RH (2010). Health disparities: a primer for public health social workers. Soc Work Public Health.

[2] Juarez PD, Matthews-Juarez P, Hood DB (2014). The public health exposome: a population-based, exposure science approach to health disparities research. Int J Environ Res Public Health.

[3] Agurs-Collins T, Alvidrez J, ElShourbagy Ferreira S (2024). Perspective: nutrition health disparities framework: a model to advance health equity. Adv Nutr.

[4] Livingston V, Jackson-Nevels B, Brown-Meredith E (2025). Poverty, allostasis, and chronic health conditions: Health disparities across the lifespan. Encyclopedia.

[5] Ampofo J, Bentum-Micah G, Xusheng Q, Sun B, Mensah Asumang R (2025). Exploring the role of teacher empathy in student mental health outcomes: a comparative SEM approach to understanding the complexities of emotional support in educational settings. Front Psychol.

[6] Maggi S, Ostry A, Callaghan K, Hershler R, Chen L, D'Angiulli A, Hertzman C (2010). Rural-urban migration patterns and mental health diagnoses of adolescents and young adults in British Columbia, Canada: a case-control study. Child Adolesc Psychiatry Ment Health.

[7] Subica AM, Link BG (2022). Cultural trauma as a fundamental cause of health disparities. Soc Sci Med.

[8] Beard S, Freeman K, Velasco ML (2024). Racism as a public health issue in environmental health disparities and environmental justice: working toward solutions. Environ Health.

[9] Kara H, Karakaya D (2026). Substance use disorders in adolescents: a qualitative systematic review. J Adv Nurs.

[10] Daniel M, Cargo MD, Lifshay J, Green LW (2004). Cigarette smoking, mental health and social support: data from a northwestern First Nation. Can J Public Health.

[11] Maghawry HF, Darwish AM, Mohammed NA (2024). Assessing emotional intelligence domains and levels in substance use disorders. Egypt J Neurol Psychiatry Neurosurg.

[12] Jayathilake A, Hodgson K, Mansoor M, Markel K, Bardwell G (2025). The potential use of digital technologies to enhance opioid agonist treatment in rural and remote communities of British Columbia, Canada. Harm Reduct J.

[13] Peng P, Chen Z, Ren S (2025). Trajectory of internet gaming disorder among Chinese adolescents: course, predictors, and long-term mental health outcomes. J Behav Addict.

[14] DelRosso LM (2025). Global perspectives on sleep health: Definitions, disparities, and implications for public health. Brain Sci.

[15] Bardwell G, Bowles JM, Mansoor M, Werb D, Kerr T (2023). Access to tablet injectable opioid agonist therapy in rural and smaller urban settings in British Columbia, Canada: a qualitative study. Subst Abuse Treat Prev Policy.

[16] Hodgson K, Bowles JM, Mansoor M, Rooke E, Bardwell G (2024). 'I'm on the coast and I'm on methadone': a qualitative study examining access to opioid agonist treatment in rural and coastal British Columbia. Can J Rural Med.

[17] Urbanoski KA, van Roode T, Selfridge M (2024). Access and barriers to safer supply prescribing during a toxic drug emergency: a mixed methods study of implementation in British Columbia, Canada. Subst Abuse Treat Prev Policy.

[18] Tsang VW, Lin MC, Huang N (2025). Compass-Canada's first child psychiatry access program: implementation and lessons learned. PLoS One.

[19] Agboji A, Freeman S, Banner D, Armstrong J, Martin-Khan M, Freeman-Idemilih A (2025). Exploring the impact of a digital reading program on apathy among community-dwelling older adults in rural Canada: insights from socioemotional selectivity theory. Geriatrics (Basel).

[20] Mitchell-Foster SM, Emon CE, Brouwer M, Elder LD, King J (2021). Disconnected perspectives: patient and care provider's experiences of substance use in pregnancy. Int J Gynaecol Obstet.

[21] Odoms-Young A, Brown AG, Agurs-Collins T, Glanz K (2024). Food insecurity, neighborhood food environment, and health disparities: State of the science, research gaps and opportunities. Am J Clin Nutr.

[22] Njoku A, Wakeel F (2019). Infusing health disparities awareness into public health curricula at a rural Midwestern university. Pedagogy Health Promot.

[23] Price JH, Khubchandani J, Webb FJ (2018). Poverty and health disparities: What can public health professionals do?. Health Promot Pract.

[24] Kitt-Lewis E, Adam MT (2025). Nurses' experiences and perspectives caring for people with substance use disorder and their families: a qualitative descriptive study. Int J Ment Health Nurs.

[25] Thekkumkara SN, Jagannathan A, Muliyala KP, Murthy P (2022). Psychosocial interventions for prisoners with mental and substance use disorders: a systematic review. Indian J Psychol Med.

[26] Page MJ, McKenzie JE, Bossuyt PM (2021). The PRISMA 2020 statement: an updated guideline for reporting systematic reviews. BMJ.

[27] Vowels LM, Carnelley KB, Stanton SC (2022). Attachment anxiety predicts worse mental health outcomes during COVID-19: Evidence from two studies. Pers Individ Dif.

[28] Hutchison A, Urbanoski K, Hore D, Wallace B (2025). Implementing community drug checking in smaller urban communities: a qualitative study exploring contextual factors to consider. Harm Reduct J.

[29] Jenkins EK, Slemon A, Haines-Saah RJ (2017). Developing harm reduction in the context of youth substance use: insights from a multi-site qualitative analysis of young people's harm minimization strategies. Harm Reduct J.

[30] Sullivan E, Cameron A, Kornelsen J (2022). Rural residents' perinatal experiences during the initial months of the COVID-19 pandemic: a qualitative study in British Columbia. J Midwifery Womens Health.

[31] Hards A, Cameron A, Sullivan E, Kornelsen J (2022). Actualizing community-academic partnerships in research: a case study on rural perinatal peer support. Res Involv Engagem.

[32] Freeman S, Martin J, Nash C (2020). Use of a digital storytelling workshop to foster development of intergenerational relationships and preserve culture with the Nak'azdli First Nation: findings from the Nak'azdli Lha'hutit'en Project. Can J Aging.

[33] Wells GA, Shea B, O'Connell D, Peterson J, Welch V, Losos M, Tugwell P The Newcastle-Ottawa Scale (NOS) for assessing the quality of nonrandomised studies in meta-analyses. http://www.ohri.ca/programs/clinical_epidemiology/oxford.asp.

[34] (2018). CASP Qualitative Studies Checklist. https://casp-uk.net/casp-tools-checklists/qualitative-studies-checklist/.

